# Diaqua­(triethano­lamine)copper(II) sulfate monohydrate

**DOI:** 10.1107/S1600536809026166

**Published:** 2009-07-11

**Authors:** Hong-Xu Guo, Sen-Ke Huang, Xi-Zhong Li

**Affiliations:** a Department of Chemistry and Environmental Science, Zhangzhou Normal University, Zhangzhou, Fujian 363000, People’s Republic of China

## Abstract

The asymmetric unit of the title compound, [Cu(C_6_H_15_NO_3_)(H_2_O)_2_]SO_4_·H_2_O, contains a complex cation, a sulfate anion and one uncoordinated water mol­ecule. In the complex cation, the Cu^II^ ion is coordinated by five O atoms (three of which are from the triethano­lamine ligand and two from coordinated water mol­ecules) and one N atom of the triethano­lamine ligand in a typical Jahn–Teller-distorted octa­hedral geometry. Classical inter­molecular O—H⋯O hydrogen bonds link the cation, the sulfate anion and the water mol­ecule into a two-dimensional network.

## Related literature

Metal-ion-containing supra­molecular structures can be used as zeolite-like matarials (Venkataraman *et al.*, 1995 ▶; Kepert & Rosseinsky, 1999 ▶), catalysts (Fujita *et al.*, 1994 ▶) and magnetic materials (Kahn, 1993 ▶). For related strutures, see: Guo *et al.* (2009 ▶); Haukka *et al.* (2005 ▶); Krabbes *et al.* (2000 ▶); Topcu *et al.* (2001 ▶); Ucar *et al.* (2004 ▶). For comparative bond lengths, see: Yeşilel *et al.* (2004 ▶). İçbudak *et al.* (1995 ▶).
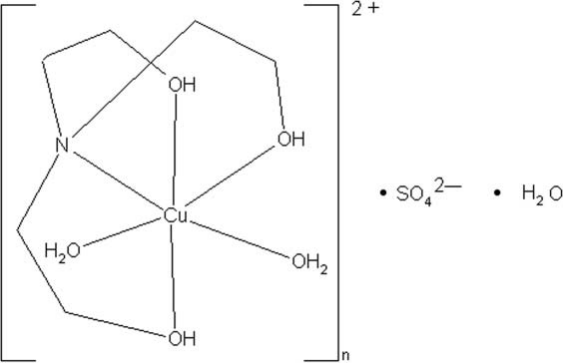


## Experimental

### 

#### Crystal data

[Cu(C_6_H_15_NO_3_)(H_2_O)_2_]SO_4_·H_2_O
*M* *_r_* = 362.84
Orthorhombic, 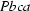

*a* = 12.502 (3) Å
*b* = 14.835 (3) Å
*c* = 15.049 (3) Å
*V* = 2791.1 (10) Å^3^
*Z* = 8
Mo *K*α radiation
μ = 1.76 mm^−1^
*T* = 293 K
0.46 × 0.43 × 0.28 mm

#### Data collection

Siemens SMART CCD area-detector diffractometer
Absorption correction: multi-scan (*SADABS*; Sheldrick, 1996 ▶) *T*_min_ = 0.471, *T*_max_ = 0.619
24803 measured reflections
3180 independent reflections
2903 reflections with *I* > 2σ(*I*)
*R*_int_ = 0.036

#### Refinement

*R*[*F* ^2^ > 2σ(*F* ^2^)] = 0.030
*wR*(*F* ^2^) = 0.095
*S* = 1.01
3180 reflections
200 parameters
14 restraints
H atoms treated by a mixture of independent and constrained refinement
Δρ_max_ = 0.66 e Å^−3^
Δρ_min_ = −0.54 e Å^−3^

### 

Data collection: *SMART* (Siemens, 1994 ▶); cell refinement: *SAINT* (Siemens, 1994 ▶); data reduction: *SAINT*; program(s) used to solve structure: *SHELXTL* (Sheldrick, 2008 ▶); program(s) used to refine structure: *SHELXTL*; molecular graphics: *SHELXTL*; software used to prepare material for publication: *SHELXTL*.

## Supplementary Material

Crystal structure: contains datablocks I, global. DOI: 10.1107/S1600536809026166/fj2232sup1.cif

Structure factors: contains datablocks I. DOI: 10.1107/S1600536809026166/fj2232Isup2.hkl

Additional supplementary materials:  crystallographic information; 3D view; checkCIF report

## Figures and Tables

**Table 1:** Hydrogen-bond geometry (Å, °)

*D*—H⋯*A*	*D*—H	H⋯*A*	*D*⋯*A*	*D*—H⋯*A*
O1*W*—H1*WA*⋯O8^i^	0.871 (9)	1.899 (13)	2.753 (3)	166 (3)
O1—H1*C*⋯O6^ii^	0.800 (10)	1.950 (11)	2.744 (2)	172 (3)
O1*W*—H1*WB*⋯O9^iii^	0.851 (10)	1.928 (12)	2.772 (3)	171 (4)
O2—H2*C*⋯O7^i^	0.788 (10)	1.992 (11)	2.775 (2)	172 (4)
O3—H3*C*⋯O6	0.810 (10)	1.822 (13)	2.609 (2)	164 (3)
O4—H4*C*⋯O9^ii^	0.825 (15)	1.932 (17)	2.750 (2)	172 (3)
O4—H4*D*⋯O1*W*	0.762 (13)	1.867 (15)	2.608 (3)	164 (3)
O5—H5*D*⋯O7	0.851 (16)	1.834 (18)	2.644 (2)	158 (3)

Symmetry codes: (i) 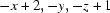; (ii) 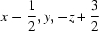; (iii) 
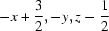
.

**Fig. 1.**
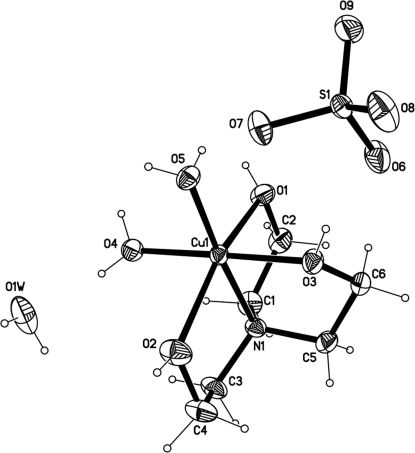
View of the structure of compound (I), showing the atom-labelling scheme. Displacement ellipsoids are drawn at the 35% probability level; H-atoms are shown as small spheres of arbitrary radius.

**Fig. 2.**
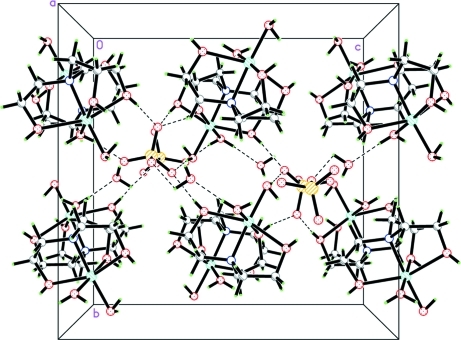
View of the 2-D hydrogen-bonded network in the packing of the title compound. The packing is viewed along the a axis; O-H···O interactions are shown as dashed lines.

## supplementary crystallographic information

### Comment

Many workers from a variety of scientific disciplines are interested in the crystal design and engineering of multidimensional arrays and networks containing metal ions as nodes. Metal-ion-containing supramolecular structures can be used as zeolite-like matarials (Venkataraman *et al.*, 1995; Kepert & Rosseinsky, 1999), catalysts (Fujita *et al.*, 1994) or magnetic materials (Kahn, 1993). Triethanolamine(TEA)is a good potential ligand to the incorporation of metals into metal-ion-containing supramolecular framework, and many compounds constructed from TEA have been reported in the last decade (Krabbes *et al.*, 2000; Topcu *et al.*, 2001; Ucar *et al.*, 2004; Haukka *et al.*, 2005;Guo *et al.*, 2009). In this work, we employed TEA and CuSO_4_ for producing a novel complex, [Cu(C_6_H_15_NO_3_)(H_2_O)_2_].SO_4_.H_2_O(I).

A view of (I) and its numbering scheme are shown in Fig. 1. The crystal structure consists of a complex cation, one sulfate anion and one lattice water molecue. In the complex cation, the Cu^II^ ion is coordinated by five O atoms, in which three from the TEA ligand and two from coordination water molecules, and one N atom of the TEA ligand in a highly distorted octahedral configuration of the CuNO5 type, in which The Cu—O bond lengths and O—Cu—N bond angles are in the range of 1.944 (2)–2.389 (2) Å, 80.30 (7)–175.98 (7)°, respectively. and the Cu—N bond length is of 2.033 (2) Å, which is similar to that of the other related compounds(İçbudak *et al.*, 1995; Yeşilel, *et al.*, 2004). The neutral TEA ligand behaves as a tetradentate ligand using all the donor sites (N1, O1, O2 and O3).

In the crystal structure of (I), classical intermolecular O—H···O hydrogen bonds are observed (Table 2), which link the hydroxies, coordinated water molecues of the cation, sulfate anion and lattice water molecue into a two-dimensional hydrogen-bonded network and stabilize the crystal packing (Fig. 2).

### Experimental

CuSO_4_.5H_2_O (0.5002 g, 2 mmol) was dissolved in 10 ml water and the pH was adjusted to 8 with triethanolamine. Blue crystals of (I) separated from the filtered solution at room temperature overseveral days.

### Refinement

All H atoms bound to carbon were refined using a riding model with C—H = 0.97Å and *U*_iso_(H) = 1.2*U*_eq_(C).Three hydroxy H atoms were located in a difference map and refined with O—H distance restraints of 0.80 (1) A and with *U*_iso_(H) = 1.5*U*_eq_(O).The two coordinated water H atoms were located in a difference map and refined with O—H and H···H distance restraints of 0.85 (1) and 1.39 (1) Å, respectively, and with *U*_iso_(H) = 1.2*U*_eq_(O), while the lattice water H atoms were located in a difference map and refined with O—H and H···H distance restraints of 0.84 (1) and 1.39 (1) Å, respectively, and with *U*_iso_(H) = 1.2*U*_eq_(O).

### Crystal data

**Table:**

[Cu(C_6_H_15_NO_3_)(H_2_O)_2_]SO_4_·H_2_O	*F*(000) = 1512
*M**_r_* = 362.84	*D*_x_ = 1.727 Mg m^−^^3^
Orthorhombic, *P**b**c**a*	Mo *K*α radiation, λ = 0.71073 Å
Hall symbol: -P 2ac 2ab	Cell parameters from 24803 reflections
*a* = 12.502 (3) Å	θ = 3.1–27.4°
*b* = 14.835 (3) Å	µ = 1.76 mm^−^^1^
*c* = 15.049 (3) Å	*T* = 293 K
*V* = 2791.1 (10) Å^3^	Block, blue
*Z* = 8	0.46 × 0.43 × 0.28 mm

### Data collection

**Table:**

Siemens SMART CCD area-detector diffractometer	3180 independent reflections
Radiation source: fine-focus sealed tube	2903 reflections with *I* > 2σ(*I*)
graphite	*R*_int_ = 0.036
ω scans	θ_max_ = 27.4°, θ_min_ = 3.1°
Absorption correction: multi-scan (*SADABS*; Sheldrick, 1996)	*h* = −16→15
*T*_min_ = 0.471, *T*_max_ = 0.619	*k* = −17→19
24803 measured reflections	*l* = −19→19

### Refinement

**Table:**

Refinement on *F*^2^	Secondary atom site location: difference Fourier map
Least-squares matrix: full	Hydrogen site location: inferred from neighbouring sites
*R*[*F*^2^ > 2σ(*F*^2^)] = 0.030	H atoms treated by a mixture of independent and constrained refinement
*wR*(*F*^2^) = 0.095	*w* = 1/[σ^2^(*F*_o_^2^) + (0.062*P*)^2^ + 2.0253*P*] where *P* = (*F*_o_^2^ + 2*F*_c_^2^)/3
*S* = 1.01	(Δ/σ)_max_ = 0.001
3180 reflections	Δρ_max_ = 0.66 e Å^−^^3^
200 parameters	Δρ_min_ = −0.53 e Å^−^^3^
14 restraints	Extinction correction: *SHELXTL* (Sheldrick, 2008), Fc^*^=kFc[1+0.001xFc^2^λ^3^/sin(2θ)]^-1/4^
Primary atom site location: structure-invariant direct methods	Extinction coefficient: 0.0166 (8)

### Special details

**Table:**

Geometry. All e.s.d.'s (except the e.s.d. in the dihedral angle between two l.s. planes) are estimated using the full covariance matrix. The cell e.s.d.'s are taken into account individually in the estimation of e.s.d.'s in distances, angles and torsion angles; correlations between e.s.d.'s in cell parameters are only used when they are defined by crystal symmetry. An approximate (isotropic) treatment of cell e.s.d.'s is used for estimating e.s.d.'s involving l.s. planes.
Refinement. Refinement of *F*^2^ against ALL reflections. The weighted *R*-factor *wR* and goodness of fit *S* are based on *F*^2^, conventional *R*-factors *R* are based on *F*, with *F* set to zero for negative *F*^2^. The threshold expression of *F*^2^ > σ(*F*^2^) is used only for calculating *R*-factors(gt) *etc*. and is not relevant to the choice of reflections for refinement. *R*-factors based on *F*^2^ are statistically about twice as large as those based on *F*, and *R*- factors based on ALL data will be even larger.

### Fractional atomic coordinates and isotropic or equivalent isotropic displacement parameters (Å^2^)

**Table:**

	*x*	*y*	*z*	*U*_iso_*/*U*_eq_	
Cu1	0.817285 (19)	0.161186 (16)	0.563698 (16)	0.02242 (12)	
N1	0.79877 (14)	0.28534 (12)	0.50841 (12)	0.0263 (4)	
O1	0.72865 (15)	0.24227 (11)	0.67944 (11)	0.0355 (4)	
H1C	0.6857 (19)	0.2171 (19)	0.7100 (18)	0.043*	
O2	0.87331 (18)	0.13070 (13)	0.42137 (12)	0.0449 (4)	
H2C	0.902 (2)	0.0986 (19)	0.3874 (18)	0.054*	
O3	0.96021 (12)	0.20915 (11)	0.59952 (11)	0.0327 (4)	
H3C	0.992 (2)	0.1803 (16)	0.6366 (15)	0.039*	
O4	0.67091 (12)	0.11906 (11)	0.52784 (11)	0.0303 (3)	
H4C	0.636 (2)	0.0983 (19)	0.5697 (12)	0.036*	
H4D	0.668 (2)	0.0999 (17)	0.4809 (10)	0.036*	
O5	0.84098 (12)	0.04673 (10)	0.62306 (11)	0.0295 (3)	
H5C	0.8032 (16)	0.0406 (13)	0.6761 (12)	0.035*	
H5D	0.9054 (12)	0.0446 (18)	0.6411 (16)	0.035*	
O6	1.07432 (17)	0.14707 (12)	0.73118 (14)	0.0482 (5)	
O7	1.02862 (13)	−0.00389 (11)	0.68697 (11)	0.0368 (4)	
O8	1.20422 (14)	0.02931 (15)	0.74339 (13)	0.0488 (5)	
O9	1.05504 (15)	0.03349 (14)	0.84170 (11)	0.0474 (5)	
C1	0.6991 (2)	0.32729 (17)	0.54436 (19)	0.0353 (5)	
H1A	0.6376	0.2939	0.5228	0.042*	
H1B	0.6934	0.3886	0.5224	0.042*	
C2	0.6971 (2)	0.32862 (16)	0.64443 (18)	0.0368 (5)	
H2A	0.7454	0.3748	0.6661	0.044*	
H2B	0.6255	0.3432	0.6648	0.044*	
C3	0.7865 (2)	0.27310 (17)	0.41066 (15)	0.0374 (5)	
H3A	0.7908	0.3315	0.3818	0.045*	
H3B	0.7164	0.2480	0.3984	0.045*	
C4	0.8714 (3)	0.21142 (19)	0.37171 (16)	0.0453 (6)	
H4A	0.8551	0.1984	0.3100	0.054*	
H4B	0.9409	0.2405	0.3743	0.054*	
C5	0.8955 (2)	0.34057 (14)	0.52931 (17)	0.0334 (5)	
H5A	0.8742	0.4026	0.5393	0.040*	
H5B	0.9438	0.3395	0.4788	0.040*	
C6	0.95341 (18)	0.30592 (15)	0.61051 (15)	0.0323 (5)	
H6A	1.0243	0.3322	0.6145	0.039*	
H6B	0.9140	0.3209	0.6641	0.039*	
S1	1.09031 (4)	0.05082 (3)	0.75083 (3)	0.02484 (15)	
O1W	0.65014 (16)	0.0231 (2)	0.38367 (16)	0.0664 (7)	
H1WA	0.6900 (18)	−0.002 (3)	0.3429 (19)	0.080*	
H1WB	0.5855 (10)	0.012 (3)	0.370 (2)	0.080*	

### Atomic displacement parameters (Å^2^)

**Table:**

	*U*^11^	*U*^22^	*U*^33^	*U*^12^	*U*^13^	*U*^23^
Cu1	0.02221 (17)	0.02101 (17)	0.02405 (17)	−0.00087 (9)	−0.00356 (8)	0.00115 (8)
N1	0.0297 (9)	0.0254 (8)	0.0237 (8)	0.0019 (7)	−0.0029 (6)	0.0020 (7)
O1	0.0411 (9)	0.0307 (8)	0.0348 (9)	−0.0030 (7)	0.0084 (7)	0.0015 (6)
O2	0.0641 (13)	0.0379 (10)	0.0328 (9)	0.0127 (9)	0.0087 (8)	−0.0050 (7)
O3	0.0293 (8)	0.0305 (8)	0.0383 (9)	−0.0047 (6)	−0.0108 (6)	0.0063 (6)
O4	0.0286 (8)	0.0330 (9)	0.0292 (8)	−0.0045 (6)	−0.0041 (6)	−0.0027 (6)
O5	0.0237 (7)	0.0276 (8)	0.0371 (9)	0.0004 (6)	−0.0019 (6)	0.0064 (6)
O6	0.0613 (12)	0.0291 (8)	0.0541 (11)	−0.0006 (8)	−0.0309 (10)	0.0031 (8)
O7	0.0305 (8)	0.0368 (8)	0.0432 (9)	0.0059 (7)	−0.0116 (7)	−0.0122 (7)
O8	0.0234 (8)	0.0750 (13)	0.0480 (11)	0.0101 (9)	−0.0048 (7)	−0.0188 (10)
O9	0.0400 (10)	0.0720 (13)	0.0301 (9)	−0.0181 (9)	0.0007 (7)	0.0043 (8)
C1	0.0351 (12)	0.0285 (11)	0.0422 (13)	0.0086 (9)	−0.0014 (10)	−0.0001 (9)
C2	0.0402 (13)	0.0274 (11)	0.0430 (14)	0.0027 (9)	0.0075 (10)	−0.0052 (9)
C3	0.0494 (13)	0.0395 (12)	0.0232 (10)	0.0049 (11)	−0.0083 (10)	0.0048 (9)
C4	0.0582 (16)	0.0522 (15)	0.0254 (11)	0.0055 (13)	0.0059 (11)	0.0023 (10)
C5	0.0384 (13)	0.0261 (10)	0.0357 (12)	−0.0080 (9)	−0.0042 (10)	0.0078 (8)
C6	0.0334 (11)	0.0307 (11)	0.0328 (11)	−0.0092 (9)	−0.0050 (9)	0.0015 (8)
S1	0.0206 (3)	0.0285 (3)	0.0254 (3)	0.00207 (19)	−0.00341 (16)	−0.00080 (18)
O1W	0.0345 (10)	0.1096 (19)	0.0551 (13)	−0.0124 (12)	0.0036 (9)	−0.0468 (13)

### Geometric parameters (Å, °)

**Table:**

Cu1—O5	1.9414 (15)	O7—S1	1.4755 (16)
Cu1—O3	1.9975 (16)	O8—S1	1.4638 (18)
Cu1—O4	2.0076 (16)	O9—S1	1.4596 (18)
Cu1—N1	2.0343 (18)	C1—C2	1.506 (4)
Cu1—O2	2.2984 (18)	C1—H1A	0.9700
Cu1—O1	2.3893 (17)	C1—H1B	0.9700
N1—C3	1.490 (3)	C2—H2A	0.9700
N1—C5	1.494 (3)	C2—H2B	0.9700
N1—C1	1.495 (3)	C3—C4	1.519 (4)
O1—C2	1.440 (3)	C3—H3A	0.9700
O1—H1C	0.800 (10)	C3—H3B	0.9700
O2—C4	1.412 (3)	C4—H4A	0.9700
O2—H2C	0.788 (10)	C4—H4B	0.9700
O3—C6	1.448 (3)	C5—C6	1.510 (3)
O3—H3C	0.810 (10)	C5—H5A	0.9700
O4—H4C	0.825 (15)	C5—H5B	0.9700
O4—H4D	0.762 (13)	C6—H6A	0.9700
O5—H5C	0.931 (14)	C6—H6B	0.9700
O5—H5D	0.851 (16)	O1W—H1WA	0.871 (9)
O6—S1	1.4717 (18)	O1W—H1WB	0.851 (10)
			
O5—Cu1—O3	92.92 (7)	C2—C1—H1B	109.1
O5—Cu1—O4	89.46 (7)	H1A—C1—H1B	107.9
O3—Cu1—O4	177.25 (7)	O1—C2—C1	110.46 (19)
O5—Cu1—N1	175.92 (7)	O1—C2—H2A	109.6
O3—Cu1—N1	83.66 (7)	C1—C2—H2A	109.6
O4—Cu1—N1	93.91 (7)	O1—C2—H2B	109.6
O5—Cu1—O2	102.14 (7)	C1—C2—H2B	109.6
O3—Cu1—O2	92.81 (8)	H2A—C2—H2B	108.1
O4—Cu1—O2	88.05 (8)	N1—C3—C4	112.50 (19)
N1—Cu1—O2	80.31 (7)	N1—C3—H3A	109.1
O5—Cu1—O1	100.12 (6)	C4—C3—H3A	109.1
O3—Cu1—O1	92.23 (7)	N1—C3—H3B	109.1
O4—Cu1—O1	85.98 (7)	C4—C3—H3B	109.1
N1—Cu1—O1	77.85 (6)	H3A—C3—H3B	107.8
O2—Cu1—O1	156.89 (6)	O2—C4—C3	108.6 (2)
C3—N1—C5	110.95 (18)	O2—C4—H4A	110.0
C3—N1—C1	108.82 (18)	C3—C4—H4A	110.0
C5—N1—C1	111.76 (18)	O2—C4—H4B	110.0
C3—N1—Cu1	107.77 (14)	C3—C4—H4B	110.0
C5—N1—Cu1	108.55 (13)	H4A—C4—H4B	108.4
C1—N1—Cu1	108.89 (14)	N1—C5—C6	111.81 (17)
C2—O1—Cu1	107.96 (13)	N1—C5—H5A	109.3
C2—O1—H1C	116 (2)	C6—C5—H5A	109.3
Cu1—O1—H1C	120 (2)	N1—C5—H5B	109.3
C4—O2—Cu1	108.75 (14)	C6—C5—H5B	109.3
C4—O2—H2C	100 (3)	H5A—C5—H5B	107.9
Cu1—O2—H2C	150 (3)	O3—C6—C5	105.85 (17)
C6—O3—Cu1	109.37 (12)	O3—C6—H6A	110.6
C6—O3—H3C	118 (2)	C5—C6—H6A	110.6
Cu1—O3—H3C	116 (2)	O3—C6—H6B	110.6
Cu1—O4—H4C	113.1 (19)	C5—C6—H6B	110.6
Cu1—O4—H4D	114 (2)	H6A—C6—H6B	108.7
H4C—O4—H4D	123 (2)	O9—S1—O8	109.10 (12)
Cu1—O5—H5C	113.7 (10)	O9—S1—O6	108.55 (13)
Cu1—O5—H5D	108.9 (18)	O8—S1—O6	109.17 (13)
H5C—O5—H5D	101.7 (15)	O9—S1—O7	110.82 (11)
N1—C1—C2	112.4 (2)	O8—S1—O7	109.80 (10)
N1—C1—H1A	109.1	O6—S1—O7	109.38 (10)
C2—C1—H1A	109.1	H1WA—O1W—H1WB	107.0 (15)
N1—C1—H1B	109.1		

### Hydrogen-bond geometry (Å, °)

**Table:**

*D*—H···*A*	*D*—H	H···*A*	*D*···*A*	*D*—H···*A*
O1W—H1WA···O8^i^	0.87 (1)	1.90 (1)	2.753 (3)	166 (3)
O1—H1C···O6^ii^	0.80 (1)	1.95 (1)	2.744 (2)	172 (3)
O1W—H1WB···O9^iii^	0.85 (1)	1.93 (1)	2.772 (3)	171 (4)
O2—H2C···O7^i^	0.79 (1)	1.99 (1)	2.775 (2)	172 (4)
O3—H3C···O6	0.81 (1)	1.82 (1)	2.609 (2)	164 (3)
O4—H4C···O9^ii^	0.83 (2)	1.93 (2)	2.750 (2)	172 (3)
O4—H4D···O1W	0.76 (1)	1.87 (2)	2.608 (3)	164 (3)
O5—H5D···O7	0.85 (2)	1.83 (2)	2.644 (2)	158 (3)

Symmetry codes: (i) −*x*+2, −*y*, −*z*+1; (ii) *x*−1/2, *y*, −*z*+3/2; (iii) −*x*+3/2, −*y*, *z*−1/2.
